# Missouri State Dental Association

**Published:** 1887-07

**Authors:** William Conrad

**Affiliations:** Cor. Secretary. Hotel Beers, St. Louis, Mo.


### MISSOURI STATE DENTAL ASSOCIATION.

The twenty-third annual meeting of the Missouri State Dental Association was held at Kansas City, June 21st and 24th. The following officers were elected for 1888:

President, Dr. Wm. N. Morrison, St. Louis; 1st Vice-President, Dr. S. M. Nicholson, Fayette; 2d Vice-President, Dr. J. F. McWilliams, Mexico; Rec. Secretary, Dr. John G. Harper, St. Louis; Cor. Secretary, Dr. Wm. Conrad, St. Louis; Treasurer, Dr. James A. Price, Weston.

The next meeting will be held the first Tuesday after July 4th, 1888 at Pertel Springs, Warrensburg, Mo.

WILLIAM CONRAD,

*Cor. Secretary.*

Hotel Beers, St. Louis, Mo.

